# Ruptured Ectopic Pregnancy with an Intrauterine Device: Case Report and Sonographic Considerations

**DOI:** 10.5811/cpcem.2020.7.48258

**Published:** 2020-10-20

**Authors:** Derick D. Jones, Tobias Kummer, Jessica C. Schoen

**Affiliations:** * Mayo Clinic, Department of Emergency Medicine, Rochester, Minnesota; † Mayo Clinic Health System Albert Lea and Austin, Department of Emergency Medicine, Austin, Minnesota

**Keywords:** ectopic pregnancy, point-of-care ultrasound, intrauterine device

## Abstract

**Introduction:**

Ectopic pregnancy carries a high morbidity and mortality; patients are at risk for rupture and life-threatening hemorrhage.

**Case Report:**

We present a rare case of ruptured abdominal ectopic pregnancy in a patient with a well-positioned intrauterine device (IUD) and discuss the diagnostic utility that transabdominal point-of-care ultrasound (POCUS) can have when performed at the bedside.

**Conclusion:**

While pregnancy with an IUD in place is rare, when it is encountered the emergency provider should maintain a high degree of suspicion for extrauterine pregnancy and perform prompt evaluation for hemorrhagic shock using diagnostic POCUS.

## INTRODUCTION

In the general population, the incidence of ectopic pregnancy is estimated at 2%.^1^,^2^ However, among patients presenting to the emergency department (ED) with complaints of first-trimester abdominal pain, vaginal bleeding, or both, the prevalence of ectopic pregnancy is significantly higher, ranging from 6–16%.^1^–^4^ Ectopic pregnancy continues to confer significant maternal risk with ruptured ectopic pregnancies accounting for approximately 3% of maternal deaths.^5^

There are a variety of effective contraceptive methods available including female or male sterilization, oral contraceptive pills, long-acting reversible contraceptives, and male condoms.^6^ From 2015 to 2017, approximately 10% of women in the United States aged 15–49 who used contraception reported using long-acting reversible contraceptives (including contraceptive implants and intrauterine devices [IUD]).^6^ In the case of contraceptive failure, current IUD use significantly increases the risk for ectopic pregnancy when compared to other contraceptive methods. In a case-control study by Li at al, the risk of ectopic pregnancy was approximately four-fold higher for current oral contraceptive users and more than 20-fold higher in current IUD users compared to women currently not using contraception.^7^ Additionally, IUD use increases the risk that an ectopic pregnancy will implant at a more distal site.^8^ In their study population, Bouyer et al found that ectopic pregnancies that occurred with an IUD in place more frequently implanted in distal sites including the ovary (5%) and abdomen (2%). (Overall rates of implantation in the ovary and the abdomen were 3.2% and 1.3%, respectively.)^8^

Ruptured ectopic pregnancy is potentially life threatening. Therefore, the emergency provider needs to maintain a high index of suspicion in the right clinical setting. Point-of-care ultrasound (POCUS) is commonly used to assist in the diagnosis and management of a variety of conditions, including ectopic pregnancy.^9^–^12^ Here, we present a case of a ruptured abdominal ectopic pregnancy in a 21-year-old female with an IUD, diagnosed by POCUS in the ED.

## CASE REPORT

A healthy 21-year-old female presented to the local community ED for evaluation of syncope and abdominal pain. She had been evaluated at the local urgent care three days prior, complaining of constipation and was discharged with a prescription for lactulose. The following day she developed abdominal cramping and several near-syncopal spells. The day of presentation, the patient took a dose of lactulose and then developed sudden onset diarrhea and worsening abdominal pain. While seated on the toilet, she suffered a brief syncopal episode without associated trauma. She reported that her last menstrual period was one month prior and that she had a levonorgestrel IUD in place for contraception. She reported no fever, vomiting, or vaginal bleeding.

On arrival in the ED, the patient was awake, alert, and in no distress. Vital signs included a temperature of 36.5º Celsius, heart rate of 84 beats per minute, blood pressure of 78/64 millimeters of mercury (mm Hg), respiratory rate of 18 breaths per minute, and oxygen saturation of 100% on room air. On examination, her abdomen was soft and mildly distended with diffuse tenderness to palpation; she had no guarding. The patient was maintained on a cardiac monitor, and a peripheral intravenous line was established. She was resuscitated with one liter normal saline bolus and her blood pressure improved to 101/55 mm Hg. Initial laboratory evaluation revealed anemia: hemoglobin 7.9 grams (g) per deciliter (dL) (reference [ref] range: 12.0–15.5 g/dL) and hematocrit 23.4% (ref range: 34.9–44.5%). Her electrolytes, blood glucose, and lactate were unremarkable.

**Image 1:**
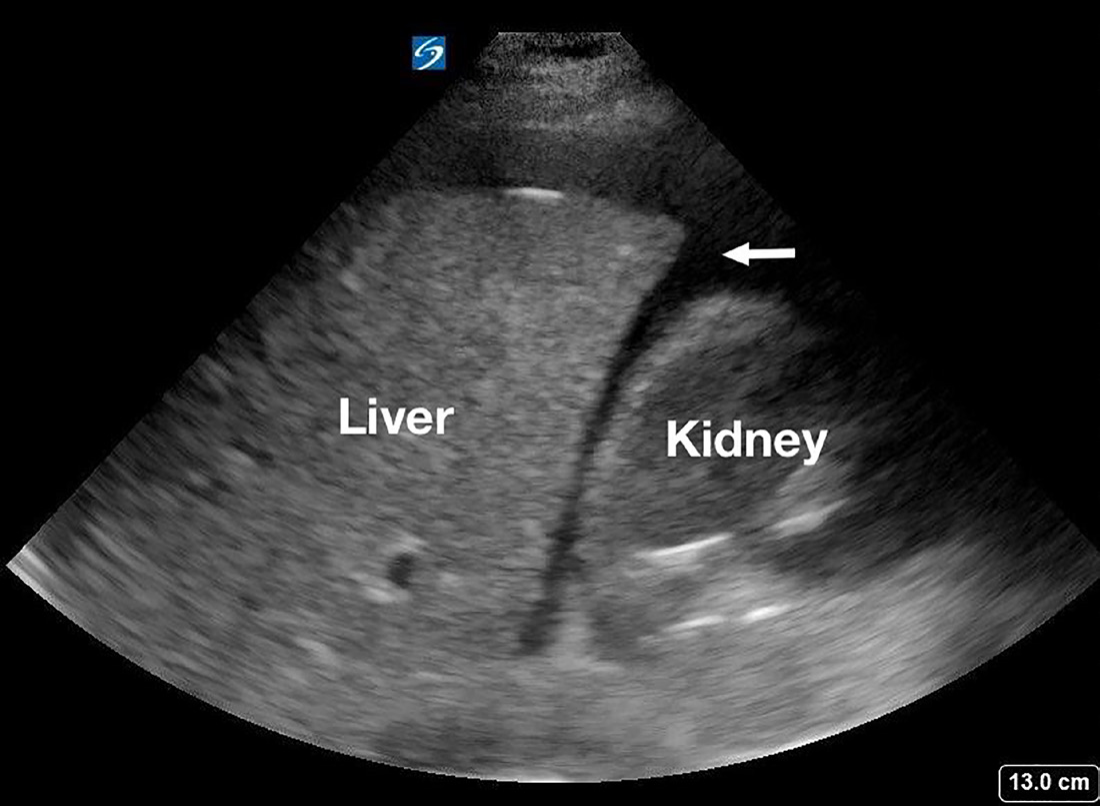
Point-of-care ultrasound of the right upper quadrant of the abdomen demonstrating free fluid in Morison’s pouch (arrow).

**Image 2:**
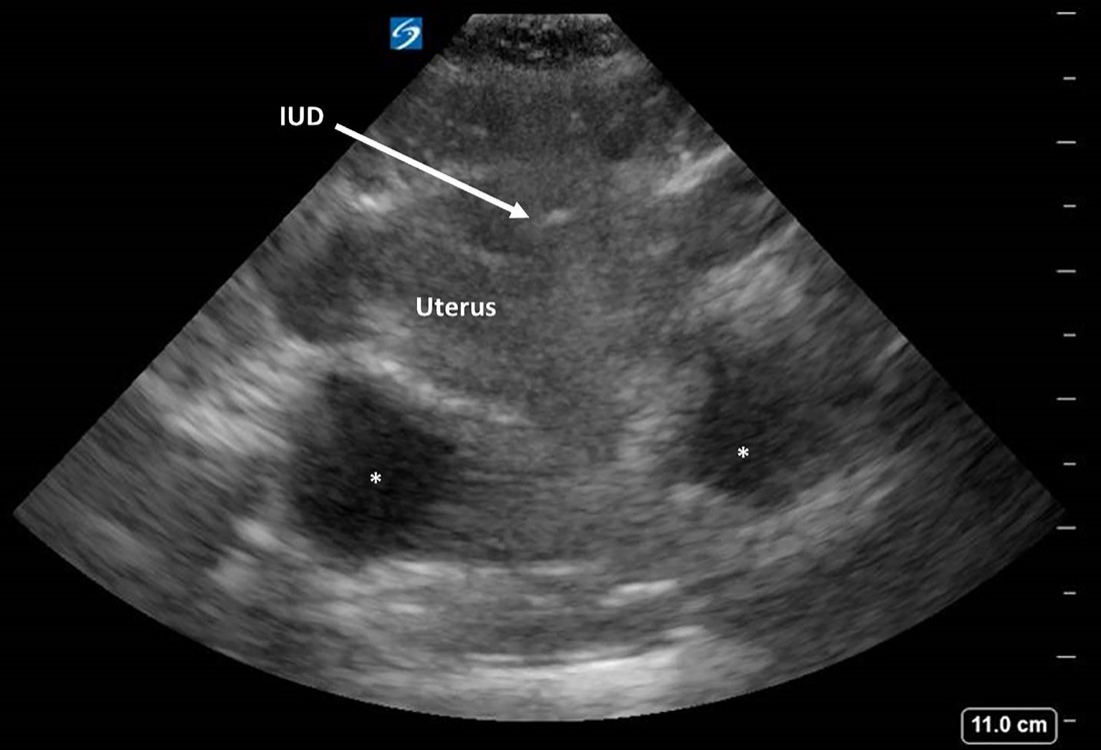
Transabdominal point-of-care ultrasound of the pelvis demonstrating an intrauterine device within the uterus (arrow), no evidence of intrauterine pregnancy, and free fluid posterior to the uterus (asterisks).

The patient’s abdominal pain, unexplained anemia, and history of syncope raised concern for hemoperitoneum. POCUS was notable for free fluid in the hepatorenal recess (Morison’s pouch) in the right upper quadrant (Image 1). Transabdominal point-of-care pelvic ultrasound demonstrated an IUD, but no visible intrauterine pregnancy (IUP) (Image 2). Subsequently, the urine pregnancy test result returned and was positive.

**Image 3:**
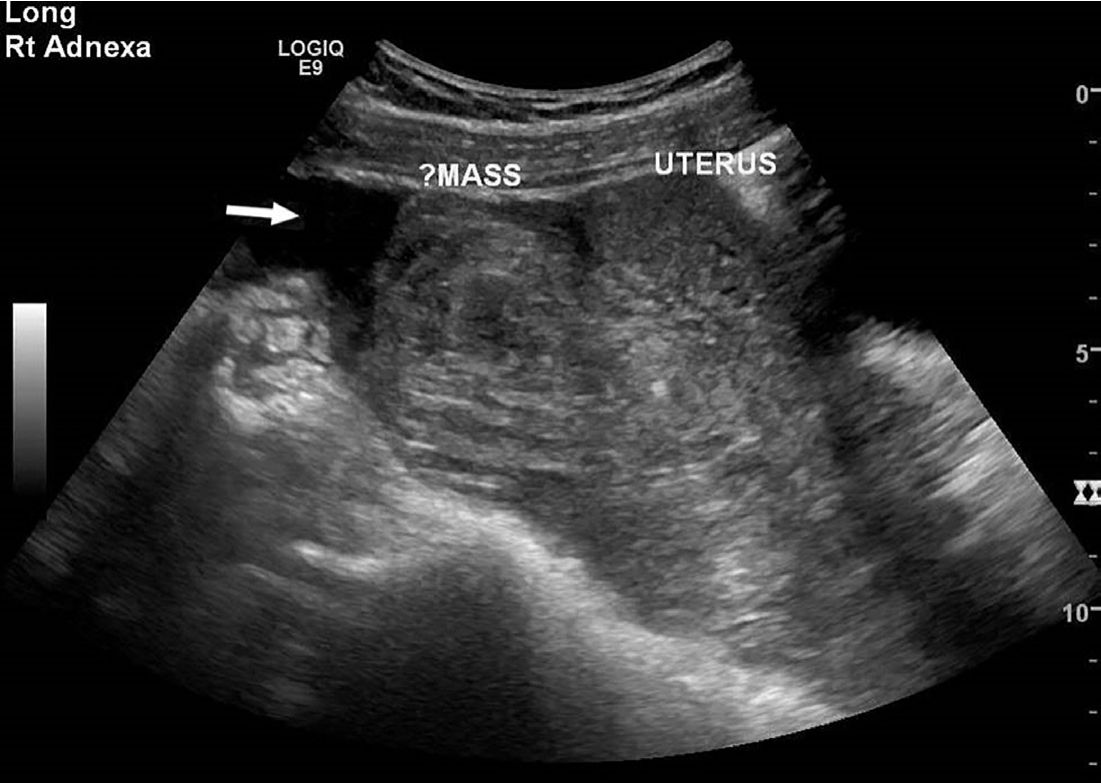
Short axis. Transvaginal ultrasound demonstrating a 10-centimeter heterogeneous mass posterior to the uterus, moderate free fluid in the pelvis (arrow), and no evidence of intrauterine pregnancy or adnexal abnormality.

The obstetric physician on-call was consulted, evaluated the patient in the ED, reviewed the POCUS images, and requested comprehensive transvaginal (TV) ultrasound (Image 3). This demonstrated a 10-centimeter heterogeneous mass posterior to the uterus, moderate free fluid in the pelvis, and no evidence of intrauterine pregnancy (IUP) or adnexal abnormality. The IUD was noted to be in good position in the endometrial canal. In consultation with the obstetrician, because these findings raised concern for alternate pathology (such as malignancy) that might require subspecialty care, the decision was made to transfer the patient to the regional tertiary care center for definitive management. Repeat hemoglobin was 6.2 g/dL (ref range: 12.0–15.5 g/dL). Transfusion with one unit of packed red blood cells was initiated, and she was transferred via air ambulance.

CPC-EM Capsule

What do we already know about this clinical entity?

*Given the significant morbidity and mortality associated with ectopic pregnancy, a high index of suspicion must be maintained. The diagnosis can be challenging*.

What makes this presentation of disease reportable?

*We present a rare case of ruptured abdominal ectopic pregnancy in a patient with a well-positioned intrauterine device*.

What is the major learning point?

*When ectopic pregnancy is suspected, an empty uterus and free fluid in Morison’s pouch visualized with ultrasound are highly specific for ruptured ectopic pregnancy*.

How might this improve emergency medicine practice?

*Performing diagnostic point-of-care ultrasound when there is suspicion for extrauterine pregnancy can decrease unnecessary or dangerous delays in treatment*.

On arrival to the referral ED, she was hemodynamically stable with a blood pressure of 119/54 mm Hg and a heart rate of 82 beats per minute. She was evaluated by gynecologic surgery and promptly taken to the operating room. She was noted to have extensive hemoperitoneum and organized clot posterior to the uterus. Once removed, the site of the ectopic pregnancy appeared to be abdominal in the posterior cul de sac medial to the left uterosacral ligament and lateral to the rectum. This was excised and later confirmed by pathology. The patient tolerated the procedure well and was discharged later that day. Because of the abdominal location of the ectopic pregnancy, she was treated with intramuscular methotrexate and followed until beta human gonadotropin (hCG) levels were negative approximately four weeks later.

## DISCUSSION

Because of the significant morbidity and mortality associated with ectopic pregnancy, a high index of suspicion must be maintained. The diagnosis, however, can be challenging. Risk factors for ectopic pregnancy include previous ectopic pregnancy, previous tubal surgery, documented tubal pathology, in utero diethylstilbestrol exposure, previous genital infection such as pelvic inflammatory disease, infertility, a history of smoking, and age greater than 35 years; however, ectopic pregnancy frequently occurs in women with no known risk factors.^1^,^2^,^13^ Additionally, the diagnosis cannot be reliably confirmed or excluded based on history or physical exam findings alone.^1^,^3^,^4^,^13^

Many patients present before rupture and can be diagnosed rapidly with a combination of quantitative serum hCG test and POCUS.^1^,^2^,^13^ Transvaginal ultrasound is the diagnostic imaging modality of choice^1^,^2^,^13^ and is highly sensitive (87–99%) and specific (94–99.9%).^13^ Although TV ultrasound is the preferred imaging modality, it is not readily available in all EDs, especially in non-academic or rural settings. In the absence of TV ultrasound, transabdominal pelvic ultrasound can be sufficient to rule out ectopic pregnancy when an IUP is identified.^9^ While the gestational sac is the earliest sign of an IUP, less experienced practitioners should consider using a visible yolk sac as a more definitive sign of an IUP. A pseudogestanional sac can be seen in ectopic pregnancy and mimic the gestational sac of a normal pregnancy.^2^,^13^

Classically, patients with ruptured ectopic pregnancy present with signs of shock (eg, tachycardia, hypotension),^2^ but the degree of hemodynamic instability can be variable.^1^,^10^ In one study, only 12% of patients with confirmed ruptured ectopic pregnancy presented with tachycardia and hypotension.^10^ Hemodynamically unstable patients or those with signs of intraperitoneal bleeding require operative intervention for definitive management.^1^,^2^

When an ectopic pregnancy is suspected, the presence of free fluid in the right upper quadrant (Morison’s pouch) noted on POCUS should raise the suspicion of a ruptured ectopic pregnancy with hemorrhage.^10^,^11^ Early identification of hemoperitoneum reduces the time to diagnosis and operative management when compared to patients evaluated with consultative pelvic ultrasound performed by other imaging specialists.^10^ In some cases, the ectopic pregnancy may be visible on transabdominal ultrasound and can confirm the diagnosis at the bedside.^12^ A quantitative beta hCG level can provide a context for the ultrasound findings but should not be used as the deciding factor to perform an ultrasound.

Abdominal ectopic pregnancies are infrequent with an estimated incidence of about 1% of ectopic pregnancies.^2^,^8^,^14^ Abdominal ectopic pregnancies can implant on the omentum, serosa, pouches surrounding the uterus and adnexa, bowel, abdominal organs, retroperitoneum, and abdominal wall.^15^ They are often misdiagnosed and are associated with high maternal morbidity and mortality. The maternal mortality rate for abdominal ectopic pregnancies is estimated at 7.7 times higher than that observed for tubal ectopic pregnancies and 90 times higher than that observed for IUPs.^14^ In the 225 cases of early (<20 weeks gestation) abdominal ectopic pregnancy reviewed by Pool et al, blood loss or hemoperitoneum occurred in 48%, blood transfusion was required in 24%, and there were seven maternal deaths (3%).^15^ As previously mentioned, in the case of contraceptive failure, current IUD use significantly increases the risk for ectopic pregnancy,^7^ and is an independent risk factor for distal implantation site, including the abdomen.^8^

We present a rare case of ruptured abdominal ectopic pregnancy in a patient with a well-positioned IUD. Because of their rarity and variable sites of implantation, abdominal ectopic pregnancies present a diagnostic challenge. This case again illustrates that when ectopic pregnancy is suspected, transabdominal POCUS performed by the emergency provider demonstrating free fluid in Morison’s pouch and no visible IUP is consistent with a diagnosis of ruptured ectopic pregnancy^10^–^12^ and urgent operative intervention is required.^2^,^11^

In this case, the lack of tubal or adnexal abnormality and the presence of a retrouterine mass on TV ultrasound raised concern for an alternate diagnosis and prompted the transfer of the patient to a tertiary referral center. It is important to consider that patients with an IUD and ectopic pregnancy are at increased risk for implantation at distal sites, and the possibility of an abdominal pregnancy must be considered. This diagnosis cannot be excluded by normal-appearing adnexa on TV ultrasound. Efforts to confirm the diagnosis with formal imaging studies may lead to potentially unnecessary or dangerous delays in treatment or patient transfer.

## CONCLUSION

Ectopic pregnancy carries high morbidity and mortality; patients are at risk for rupture and life-threatening hemorrhage. While pregnancy with an IUD in place is rare, when it is encountered the emergency provider should be highly suspicious of an extrauterine pregnancy and consider the increased risk of distal sites of implantation such as the abdomen. This should prompt the emergency provider to pay close attention to signs of hemorrhagic shock and perform diagnostic POCUS. When an ectopic pregnancy is suspected, an empty uterus and free fluid in Morison’s pouch visualized with transabdominal POCUS are highly specific for ruptured ectopic pregnancy. In conjunction with resuscitative efforts of hemorrhagic instability if present, definitive treatment with laparoscopy should be pursued as quickly as possible. Efforts to confirm the diagnosis with formal imaging studies may lead to potentially unnecessary or dangerous delays in treatment or patient transfer.
